# Ophthalmologists on Smartphones: Image-Based Teleconsultation

**DOI:** 10.22599/bioj.118

**Published:** 2019-01-09

**Authors:** Amit Mohan, Navjot Kaur, Vinod Sharma, Pradhnya Sen, Elesh Jain, Manju Gajraj

**Affiliations:** 1 Department of pediatric ophthalmology and strabismus, Sadguru netra chikitsalaya and postgraduate institute of ophthalmology, Jankikund, Chitrakoot (MP), IN; 2 Global hospital institute of ophthalmology, Abu Road (Rajasthan), IN; 3 Sadguru Netra chikitsalya and postgraduate institute of ophthalmology, Jankikund, Chitrakoot, IN; 4 Department of ophthalmology, SMS Medical College, Jaipur, (Rajasthan), IN

**Keywords:** smartphone, telemedicine, teleophthalmology

## Abstract

**Background:**

Teleophthalmology has the potential to facilitate wider access to expert advice. It includes viewing of ophthalmic images by experts either on handheld devices like smartphones/tablets or office devices such as computer screens. However, to ensure rapid feedback, the turnaround time of any consultation must be kept to a minimum which requires use of handheld user-friendly devices. The purpose of this study was to assess whether images of different eye ailments viewed on smartphones and tablets are of comparable subjective quality as those viewed on a computer screen.

**Methods:**

This was a prospective study comparing the subjective quality of images on a smartphone, tablet and computer screen. Thirty images were analysed – 10 of extraocular morphology, 10 of the anterior segment pathology and 10 of retinal diseases. Ten ophthalmologists participated and were instructed to rate the overall quality of each image on a 7-point Likert scale (terrible-1, poor-2, average-3, fair-4, good-5, very good-6, excellent-7).

**Results:**

Overall smartphones were found to have higher ratings of subjective image quality (5.9 ± 0.48) than images displayed on tablets (5.13 ± 0.51) and computers (5.0 ± 0.37). The images were rated ‘good’ or ‘very good’ in all (100%) of the smartphone images. Fundus images and extraocular images were rated higher than anterior segment images on the smartphone. When comparing the two handheld devices with computers, both smartphones and tablets had similar image quality (p > 0.05, not significant) to computer images. However, for extraocular diseases, smartphone (6.1 ± 0.32) had significantly better image quality and images were easier to interpret compared to images on the computer (p < 0.05).

Smartphones were rated ‘very good’ in 88.33% cases. All consultants (n = 10) were comfortable with the use of smartphone images and were already using it for teleconsultation at least three times in a month. Vision technicians reported minimum delay in getting advice when sending the images on mobile application to expert ophthalmologists.

**Conclusion:**

Smartphones can be used for teleconsultation. Subjective qualities of ophthalmic images on a smartphone are similar to those on tablets and computers. For rural communities that rely on teleconsultation, this small study provides useful evidence which may support the use of smartphones, tablets or computers for viewing ophthalmic images.

## Introduction

Telemedicine is the exchange of medical information between two different locations using electronic communication which improves patient health care. Telemedicine uses multiple applications in different devices including wireless tools, email, two-way video and smartphone-based applications. Ophthalmology is a medical specialty which may benefit from telemedicine (Ayatollahi et al. 2017; BenZion and Helveston 2007; Morse 2014).

Teleophthalmology has the potential in facilitating wider access to expert advice. However, to ensure effectiveness, the turnaround time of consultation must be kept to a minimum. One way to reduce consultation time would be for ophthalmic experts to view images received on their handheld smartphone or tablet, rather than using a computer screen.

Increasing utilisation of smartphones and rapidly growing internet access worldwide makes mobile health more widely available, including in resource-poor settings. By speeding up and facilitating access to expert advice, mobile health can contribute to effective treatment, reduced referral rates and ultimately reduced costs for both healthcare systems and patients. (Al-Hadithy & Ghosh 2013; Betjeman, Soghoian & Foran 2013). The evidence is promising regarding the usability of handheld devices (smartphones and tablets) for expert teleconsultation.

This study is concerned with ophthalmological conditions presented to vision technicians at vision centres without direct access to specialised ophthalmic consultation in resource-poor settings, specifically extra ocular diseases, anterior segment pathology and diabetic retinopathy. The aim of the study was to assess whether images viewed on handheld devices by expert ophthalmologists were of comparable subjective quality compared to when viewed on a standard computer screen.

## Material and methods

The Institutional Ethics Committee of Global Hospital Institute ofOphthalmology, Abu Road (India) on Human Subjects Research, 2015–2016, granted approval, subsequent to which the study was initiated and adhered to the tenets of the declaration of Helsinki. This was a prospective study comparing the quality of images on smartphones, tablets and computer screens. 30 images were analysed- 10 of extra ocular diseases, 10 of anterior segment pathology (received from our three vision centers located in remote areas of south west India) and 10 of retinal diseases (fundus images received from community hospitals adopted in the Queen Elizabeth foundation project for diabetic retinopathy screening in district Pali). Completed consent forms were obtained from all the patients whose images were used for analysis before sending them to the participant ophthalmologists.

Three different display devices which are commonly used by ophthalmologists to view images were selected: a laptop computer screen (model HP pavilion dv4 windows 7 15.6 inches (39.62 cm)1366 × 768 pixels) used as the reference, a tablet (model Samsung Galaxy Tab 3) and a smartphone (model Apple iPhone 5S). All ophthalmologists used only the aforementioned devices for the purpose of the study, and not their own devices.

Clinical images of extraocular diseases were captured with normal digital camera (Sony DSC HX60V), anterior segment diseases by slit lamp camera (Topcon SL-D2 imaging system) and retinal images by fundus camera (TRC-NW8 Topcon Medical Systems). All of the images were sent to 10 ophthalmologists on their WhatsApp instant messaging app and registered email addresses for expert opinion. WhatsApp images were viewed on smartphones, and email images were viewed on tablets and computers. A questionnaire was used to rate the image quality and utility of using a smartphone as a teleconsultation method. Each participant viewed the 30 images on each device and were asked to rate the overall quality of each image on a 7-point Likert scale (1 = terrible to 7 = excellent). All the images were viewed on three devices simultaneously without any randomisation. The participant ophthalmologists were instructed to focus on the quality of the images, rather than on the ability to diagnose any particular condition. They were instructed that they were allowed to zoom in on images if necessary. Participants were asked to rate the quality of the images via five image features: focus, resolution, contrast, colour and composition.

Once participants had rated all the images on each device, they were asked questions concerning image quality and how frequently they use each device to view the images for professional and teleconsultation purposes. They were also asked about the use of the device’s zoom feature during the survey and whether they would feel comfortable using the device for image-based remote consultation. Finally, the vision technicians who fed the information to the ophthalmologists were asked their opinion on ease of use and delay in receiving feedback from the ophthalmologists.

Descriptive statistical analyses were performed on categorical measurements, and results are presented in either a number (%) or mean ± standard deviation (SD) using SPSS (SPSS for Windows, Version 16.0, Chicago, SPSS Inc.). When comparing two devices (smartphone vs. computer and tablet vs. computer) the paired t-test was used to compare the means of two devices using GraphPad software. P-values < 0.05 were considered statistically significant.

## Results

Table 1 presents the demographic data of the participating ophthalmologists.

**Table 1:** Demography of participating ophthalmologists.

Variable	Ophthalmologist (n)

Gender	
Male	6 (60%)
Female	4 (40%)
Mean age, years (min–max)	37.5 (26–64)

**Figure 1:**
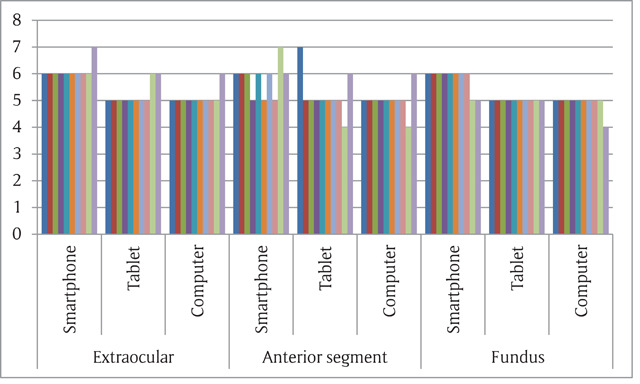
Average likert score for all the 30 images on different devices.

Mean age was 37.5 years ranging from 26 years to 64 years. 60% of the participants were male and 40% were female. Participants reported that the device used most often for professional purposes is a smartphone. All ten participants used a smartphone at least once a week for professional purposes. The computer was also used by all participants for both personal and professional purposes. However, only two ophthalmologists were using tablets. A total of nine participants reported already using their smartphone for image-based teleconsultation at least 2–3 times a month compared to two participants for computers, and one for tablet. Figure 1 shows the graphical representation of image quality of all three types of images on different devices.

Table 2 presents the results of quality of images (average of Likert scale on a 7 point scale (1 = terrible to 7 = excellent) for the smartphone, tablets and computers for all images aggregated for three different types of images (extraocular diseases, anterior segment diseases and fundus photographs).

**Table 2:** Mean quality of images on different devices.

Image category	Smartphones	Tablets	Computers

Extraocular	6.1 ± 0.32 (p = 0.02)	5.2 ± 0.42 (p = 0.84)	5.1 ± 0.32
Fundus	5.8 ± 0.63 (p = 0.12)	5.2 ± 0.79 (p = 0.69)	5.0 ± 0.47
Anterior segment	5.8 ± 0.42 (p = 0.09)	5.0 ± 0 (p = 0.88)	4.9 ± 0.32
Overall	5.9 ± 0.48 (p = 0.23)	5.13 ± 0.51 (p = 0.88)	5.0 ± 0.37

**Figure 2:**
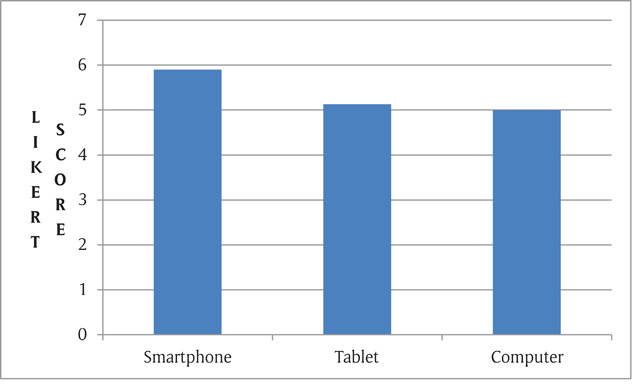
Overall average Likert scale on different devices.

Overall smartphones had higher subjective quality ratings compared to the tablets and computers. The ratings from the ophthalmologists did not differ significantly between devices. Smartphones had higher Likert scale scores for all three types of images (extraocular, anterior segment and fundus). The images were rated ‘good’ or ‘very good’ in all (100%) of the smartphone images. Fundus images and extrocular images were rated higher than anterior segment images. When comparing the two handheld devices with computers, overall both the smartphones and tablets had similar image quality (p > 0.05, not significant) as that of the computer. However, in case of extraocular diseases, the smartphone images were rated significantly better than the computer images (p < 0.05). Figure 2 shows the overall image quality on the devices.

Participants used the zoom function more often with the smartphones (n = 10) than with tablets (n = 4) and computers (n = 4). All devices had a zoom function for image viewing but most of the time clinicians were not aware about its use on tablets and computers. Almost all the participants answered that they would be confident while giving image-based consultation using the smartphones (n = 10), tablets (n = 7) and computers (n = 6).

All the vision technicians (n = 5) were comfortable with sending the images on smartphones using instant messaging apps like WhatsApp, and usually received a reply within half an hour. They reported experiencing more delays when sending the images through computer applications.

## Discussion

This small study suggests that handheld devices are a good solution for image-based teleconsultation in ophthalmic practice. Teleophthalmology is inclusion of technology by which people in remote and underserved areas can easily get specialized health care (Sharma & Rajput M 2009). Telemedicine has special significance in India because of its vast geographical spread and predominant rural population where eye care facilities are neither available nor accessible (Bedi 2009). Teleophthalmology could be useful in primary eye care, where the distance to an ophthalmologist can be a significant obstacle to diagnosis and treatment (Blomdahl & Marén 2009). In our study, vision centres are located in remote areas and they are provided expert advice through teleconsultation.

Teleophthalmology mostly adopts the store-and forward-method, followed by interactive services, such as video-conferencing. The hybrid method includes both store-and-forward and real-time communication methods. It is notable that a teleophthalmology system, like any other web application, should be designed to meet users’ specific needs and to achieve this, end-users must be involved in the design process. Otherwise, adopting the existing systems used in other communities might not be a successful approach (Sreelatha & Ramesh 2016).

Teleophthalmology utilises internet-based information technology, which allows the patient to have contact with an ophthalmic specialist at a base hospital via video conferencing. This helps the patients consult a specialist without travelling and thus bridges the gap of inaccessibility of services. In the current study, we have utilised social network services through smartphones and interpreted the clinical images, like computer-based teleconsultation, with ease.

As ophthalmology is an area in which medical imaging is important in making diagnostic decisions, eye images can be sent via telemedicine technology to facilitate making a diagnosis (John 2012). Therefore, we decided to evaluate the image quality on all the three devices. The rapid transmission of data and images is one of the most important aspects of teleophthalmology which enables doctors to consult and act very quickly. This, in turn, can improve the quality of patient care; particularly in remote areas (Bahaadinbeigy & Yogesan 2011; Yogesan, K et al. 2006). In our study, vision technicians have reported minimal delay while consulting through smartphone based instant messaging system.

A major strength of the use of smartphones is the user-friendly applications. In this study, all doctors frequently utilised the phones and were already familiar with using the zoom function on smartphones. They were less familiar with using this facility on computers and tablets. This may be a factor which influences the acceptance of smartphones in teleophthalmology.

A major application area for teleophthalmology is diabetic retinopathy, particularly for repeated screening of diabetes patients to look for signs of deterioration. Its clinical efficacy has been established (Jones & Edwards 2010) and more recently, analysis of its economic viability has been undertaken (Chasan 2014). We utilised smartphone images for a diabetic retinopathy project in this study. Other promising applications for teleophthalmology involve the support of public health screening programmes for ocular health. Fundamental acuity testing has been seen as a suitable candidate for remote delivery using smartphone-based tools (Kumar & Bulsara 2008). Recently, interest has been shown in using smartphones instead of specialised equipment wherever feasible (Zvornicanin, Zvornicanin & Hadziefendic 2014). Smartphones are becoming an inseparable part of daily life with high penetration rate all around the world: the International Telecommunication Union reported that 95.5% of the global populations are mobile subscribers in 2014 with a mobile-broadband penetration rate of 84% and 21% in developed and developing countries, respectively (Union 2014). Smartphones can be useful in fulfilling the goals of teleophthalmology by taking high-resolution photos and sending them to an expert for interpretation. Thus, not only does such technology allow diagnosis and treatment to become easier, but it also saves money and time. Thus, teleophthalmology with the aid of smartphones can prevent diseases causing blindness. Considering its ease of accessibility and its potential for utilization of highly innovative applications, the smartphone could be a promising device for teleophthalmology.

The use of mobile health in ophthalmology has allowed for greater efficiency and communications between ophthalmologist and vision technicians. As more health care providers use smartphones in the clinical setting, mobile tools have become reliable, accurate and consistent for teleconsultation. It is valuable for pictures taken for ophthalmology (Maamari 2014). Earlier studies addressed the use of tablets and smartphones in the field of radiology and emergency medicine found that tablets and smartphones were rated equal or better than computer screens (Boissin 2017; Toomey, Rainford & Leong 2014). In the current study, smartphones and tablets have similar subjective image quality (p > 0.05, not significant) as computers. Our study broadens the current knowledge on the potential for use of handheld devices in ophthalmic consultation.

## Conclusion

Handheld devices, especially smartphones, could be a substitute for computers within image-based teleconsultation in ophthalmic practice, and could save time in obtaining expert advice in resource-poor settings. Ophthalmologists receiving ophthalmic images taken in vision centres located in rural areas through ‘WhatsApp’ or email can view those images as well on a smartphone as they can on a tablet or computer, which can result in more rapid feedback. Due to the wide use of smartphones, teleophthalmology services should consider the viewing of images by experts using smartphones to be equally acceptable as viewing the images on other devices.

## References

[1] Al-Hadithy, N and Ghosh, S. 2013 Smartphones and the plastic surgeon. J Plast Reconstr Aesthetic Surg, 66: e155–61. DOI: 10.1016/j.bjps.2013.02.014 23523169

[2] Ayatollahi, H, et al. 2017 Teleophthalmology in Practice: Lessons Learned from a Pilot Project. Open Med Inform J, 11: 20–28. DOI: 10.2174/1874431101711010020 29081869 PMC5633703

[3] Bahaadinbeigy, K and Yogesan, K. 2011 Advances in teleophthalmology: Summarising published papers on teleophthalmology projects In: Advances in telemedicine: Applications in various medical disciplines and geographical regions, 231–242. Rijeka, Croatia: InTech Open DOI: 10.5772/13595

[4] Bedi, BS. 2009 Telemedicine standards: Need and Indian initiatives. Telemedicine and E-Health, 15(6): 597–599. DOI: 10.1089/tmj.2009.0061 19659417

[5] BenZion, I and Helveston, EM. 2007 Use of telemedicine to assist ophthalmologists in developing countries for the diagnosis and management of four categories of ophthalmic pathology. ClinOphthalmol, 1(4): 489–95. PMC2704545 19668527

[6] Betjeman, TJ, Soghoian, SE and Foran, MP. 2013 mHealth in Sub-Saharan Africa. Int J Telemed Appl. DOI: 10.1155/2013/482324 PMC3867872 24369460

[7] Blomdahl, S, Marén, NR and Lof, R. 2001 Tele-ophthalmology for the treatment in primary care of disorders in the anterior part of the eye. Journal of Telemedicine and Telecare, 7: 25–26. DOI: 10.1177/1357633X010070S110 11576480

[8] Boissin, C, et al. 2017 Image-based teleconsultation using smartphones or tablets: Qualitative assessment of medical experts. Emerg Med J, 34: 95–99. DOI: 10.1136/emermed-2015-205258 27707791 PMC5384429

[9] Chasan, JE, et al. 2014 Effect of a teleretinal screening program on eye care use and resources. JAMA Ophthalmol, 132(9): 1045–1051. DOI: 10.1001/jamaophthalmol.2014.1051 24875731

[10] John, S, et al. 2012 The Sankara Nethralaya mobile teleophthalmology model for comprehensive eye care delivery in rural India. Telemed J E Health, 18: 382–7. DOI: 10.1089/tmj.2011.0190 22500741

[11] Jones, S and Edwards, RT. 2010 Diabetic retinopathy screening: A systematic review of the economic evidence. Diabet Med, 27(3): 249–256. DOI: 10.1111/j.1464-5491.2009.02870.x 20536486

[12] Kumar, S, Bulsara, M and Yogesan, K. 2008 Automated determination of distance visual acuity: Towards teleophthalmology services. Clin Exp Optom, 91(6): 545–550. DOI: 10.1111/j.1444-0938.2008.00267.x 18430039

[13] Maamari, RN, et al. 2014 A mobile phone-based retinal camera for portable wide field imaging. Br J Ophthalmol, 98: 438–41. DOI: 10.1136/bjophthalmol-2013-303797 24344230

[14] Morse, A. 2014 Telemedicine in Ophthalmology: Promise and Pitfalls. Ophthalmology, 121(4): 809–811. DOI: 10.1016/j.ophtha.2013.10.033 24694522

[15] Sharma, LK and Rajput, M. 2009 Telemedicine: Socio-ethical considerations in the Indian milieu. The Medico-legal journal, 77(2): 61–65. DOI: 10.1258/rsmmlj.77.2.61 19731480

[16] Sreelatha, OK and Ramesh, SV. 2016 Teleophthalmology: Improving patient outcomes? Clin. Ophthalmol, 10: 285–295. DOI: 10.2147/OPTH.S80487 26929592 PMC4755429

[17] Toomey, RJ, Rainford, LA, Leong, DL, et al. 2014 Is the iPad suitable for image display at American Board of Radiology examinations? Am J Roentgenol, 203: 1028–33. DOI: 10.2214/AJR.13.12274 25341141

[18] Union, IT. 2014 ICT facts and figures; the world in 2014. Switzerland, Geneva.

[19] Yogesan, K, et al. 2006 Teleophthalmology. Berlin, Germany: Springer DOI: 10.1007/3-540-33714-8

[20] Zvornicanin, E, Zvornicanin, J and Hadziefendic, B. 2014 The use of smart phones in ophthalmology. Acta Inform Med, 22(3): 206–209. DOI: 10.5455/aim.2014.22.206-209 25132717 PMC4130678

